# Transient receptor potential channels’ genes forecast cervical cancer outcomes and illuminate its impact on tumor cells

**DOI:** 10.3389/fgene.2024.1391842

**Published:** 2024-05-09

**Authors:** Shan Jiang, Xuefen Lin, Qiaoling Wu, Jianfeng Zheng, Zhaolei Cui, Xintong Cai, Yanhong Li, Chaoqiang Zheng, Yang Sun

**Affiliations:** ^1^ Department of Gynecology, Clinical Oncology School of Fujian Medical University, Fujian Cancer Hospital, Fuzhou, China; ^2^ College of Integrative Medicine, Fujian University of Traditional Chinese Medicine, Fuzhou, China; ^3^ Laboratory of Biochemistry and Molecular Biology Research, Department of Clinical Laboratory, Clinical Oncology School of Fujian Medical University, Fujian Cancer Hospital, Fuzhou, China

**Keywords:** transient receptor potential channels, cervical cancer, prognosis, immunity, bioinformatics

## Abstract

**Introduction:** In recent years, there has been a strong association between transient receptor potential (TRP) channels and the development of various malignancies, drug resistance, and resistance to radiotherapy. Consequently, we have investigated the relationship between transient receptor potential channels and cervical cancer from multiple angles.

**Methods:** Patients’ mRNA expression profiles and gene variants were obtained from the TCGA database. Key genes in transient receptor potential channel prognosis-related genes (TRGs) were screened using the least absolute shrinkage and selection operator (LASSO) regression method, and a risk signature was constructed based on the expression of key genes. Various analyses were performed to evaluate the prognostic significance, biological functions, immune infiltration, and response to immunotherapy based on the risk signature.

**Results:** Our research reveals substantial differences between high and low-risk groups in prognosis, tumor microenvironment, tumor mutational load, immune infiltration, and response to immunotherapy. Patients in the high-risk group exhibited poorer prognosis, lower tumor microenvironment scores and reduced response to immunotherapy while showing increased sensitivity to specific targeted drugs. *In vitro* experiments further illustrated that inhibiting transient receptor potential channels effectively decreased the proliferation, invasion, and migration of cervical cancer cells.

**Discussion:** This study highlights the significant potential of transient receptor potential channels in cervical cancer, emphasizing their crucial role in prognostic prediction and personalized treatment strategies. The combination of TRP inhibitors with immunotherapy and targeted drugs may offer promise for individuals affected by cervical cancer.

## 1 Introduction

Cervical cancer (CC) is the predominant malignant tumor affecting the female reproductive system, with statistics showing that it accounts for 80% of all malignant tumors in this system. Additionally, there is a concerning trend towards younger individuals being diagnosed with cervical cancer (Cohen et al., 2019). In 2020, there were approximately 600,000 cases of cervical cancer diagnosed globally, resulting in 340,000 deaths (Stumbar et al., 2019; Sung et al., 2021). Despite advancements in treatment, the survival rate for patients with advanced cervical cancer remains low at around 15% due to its aggressive nature. Therefore, identifying new biomarkers for early detection and therapeutic targets is crucial for further research in this field.

Transient receptor potential (TRP) channels are a family of ion channels which involved in several physiological processes, including nociception, temperature monitoring, and sensory transduction (Nilius et al., 2007). In 1969, researchers discovered TRP channels in a subspecies of *Drosophila melanogaster*. Transient receptor potential refers to the transient calcium ion influx that occurs when the *drosophila* variety is exposed to strong light for extended periods. TRP channels can be classified into six subfamilies based on their sequence homology: TRPA (ankyrin), TRPC (canonical), TRPM (melastatin), TRPML (mucolipin), TRPP (polycystin), and TRPV (vanilloid) (Caterina and Julius, 2001; Caterina and Pang, 2016; Moore et al., 2017). The function of TRP channels in cancer has attracted more attention recently. TRP channel-related proteins expressed in various cancer cell types such as breast, prostate, lung, colon and pancreatic malignancies, have recently attracted more research attention. Specifically, TRPV6 has been shown to promote the invasion and migration of breast cancer cells (Cai et al., 2021). TRPV6 is linked to cancer cell death and proliferation in prostate cancer (Lehen kyi et al., 2007). TRPV3 has been demonstrated to facilitate cancer cell invasion and survival in lung cancer (Li et al., 2016). TRPM8 is upregulated in cancer cells and associated with a favorable prognosis in colon cancer (Pagano et al., 2023). In human pancreatic ductal adenocarcinoma tissue, TRPC1 is abundantly expressed and controls pancreatic ductal adenocarcinoma cell proliferation in a Ca^2+^ independent way (Schnipper et al., 2022). Furthermore, TRP channels are involved in the interaction between cancer cells and the tumor microenvironment. Endothelial cells express TRPC1 and TRPC6, which promote angiogenesis, the process of forming new blood vessels that supply the tumor with nutrients (Li et al., 2017; Negri et al., 2019). Additionally, TRPV1 and TRPA1 expressed by immune cells are involved in the regulation of the body’s immune response (Baral et al., 2018; Fattori et al., 2022).

Several agonists and inhibitors of the TRP pathway have been developed and tested in preclinical studies. For example, the TRPV1 antagonist capsazepine has shown some effectiveness in inhibiting the proliferation and invasion of cervical cancer cells (De La Chapa et al., 2019). Additionally, the TRPV4 selective antagonist HC-067047 has been found to induce apoptosis and limit the growth of non-small cell lung cancer cells *in vitro* (Pu et al., 2022). Waixenicin A decreased the TRPM7 protein expression and inhibited the TRPM7-like currents in GBM cells, GBM cells showed increased apoptosis and decreased proliferation, migration, invasion and survival following treatment (Wong et al., 2020). Research into the TRP pathway has the potential to open up a new frontier in oncology treatment, particularly in the development of anti-cervical cancer drugs.

In this study, we systematically assess the relationship between TRP channel-related genes (TRG) and cervical cancer and develop a reliable TRG-related prognostic signature that can be used as a validated biomarker to predict patient prognosis and immunotherapy response, offering a novel approach to tumor diagnostic and therapeutic approaches.

## 2 Materials and methods

### 2.1 Data collection

Download from the Cancer Genome Atlas (TCGA, https://tcga-data.nci.nih.gov/tcga/) and Gene Expression Omnibus (GEO, https://www.ncbi.nlm.nih.gov/geo/) cervical cancer transcriptome RNA seq data and survival information were converted to TPM format and normalized using the “SVA package” (Leek et al., 2012). In addition, copy number variation (CNV) and single nucleotide variation (SNV) were downloaded from the TCGA database. The MSigDB database and the KEGG database were used to search for TRP channel-related gene sets, and 119 genes were obtained for subsequent analysis.

### 2.2 Constructing TRG prognostic signatures

Cox regression analysis was performed to correlate TRG with CC prognosis, and TRGs associated with survival were screened out. Lasso Cox regression analysis was performed to filter prognostic TRGs and construct a prognostic signature with a score of RiskScore = Σ (Expi * Coefi) (Engebretsen and Bohlin, 2019). The CC sample was divided into high and low-risk groups according to the median division of risk values in the prognostic signature, Kaplan-Meier survival analysis was performed, ROC curves were plotted to assess predictive efficacy, and the signature was assessed using univariate and multifactorial Cox regression combining clinical factors.

### 2.3 Comprehensive analysis of TRG in terms of mutation, function, and pathway enrichment

Gene Set Variation Analysis of TRG using the “RCircos package” and the “maftools package” (Zhang et al., 2013; Mayakonda et al., 2018). GO and KEGG enrichment analyses were performed using the “clusterProfiler” package in R. The “ConsensusClusterPlus package” was used to split TRG expression into two clusters based on TRG expression in cervical cancer (Wilkerson and Hayes, 2010). The “clusterProfiler” package was used, where *p* < 0.05 and q < 0.05, indicating significant enrichment of functional annotations (Yu et al., 2012).

### 2.4 TRGs risk signature in immune cell infiltration and immunotherapy

The “CIBERSORT package” assesses the relative proportions of immune cell types according to gene expression in the samples, ESTIMATE score, and tumor purity. Sensitivity to drugs was assessed using the “pRRophetic package” (Geeleher et al., 2014).

### 2.5 Experimental materials

The cervical cancer cell lines HeLa and Siha were purchased from Meisen (Hangzhou, China) and Pricella Biotechnology Co., Ltd. (Wuhan, China). HeLa was cultured in 10% FBS, 1% Penicillin-Streptomycin in DMEM (Gibco, United States), Siha was cultured in 10% FBS, 1% Penicillin-Streptomycin in 1,640 (Gibco, United States), and both were cultured in a 37° cell incubator at saturated humidity and 5% CO2. The TRP pathway inhibitor 1-[β-(3-(4-Methoxyphenyl)propoxy)-4-methoxyphenethyl]-1H-imidazole was purchased from Sigma-Aldrich (Shanghai, China).

### 2.6 Transwell experiment

Matrigel was diluted with incomplete medium and added to the transfer chamber at 100 μL/well (at low temperature) at 37°C for 1 h. After sufficient concretion of Matrigel for the invasion assay, cell lines from the experimental and control groups were collected and digested with trypsin and added to the transfer chamber with an incomplete medium. For migration experiments, matrigel was not added. 600 μL of medium containing 10% fetal bovine serum was added to the lower chamber and incubated routinely for 24 h. The transfer chamber was removed and matrigel was wiped from the surface of the polycarbonate membrane with a cotton swab, gently washed with PBS, dried, and fixed in formaldehyde. Cells were stained with 1% crystalline violet and dried with a deionized rinse.

### 2.7 Cell wound healing assay

In cell wound healing experiments, cell lines were inoculated at 1 × 10^5^/mL in 6-well plates and after forming a monolayer of dense cells, straight lines were drawn with a 10 μL gun tip, and cell fragments were washed with PBS. After 2 consecutive days of observation, wound healing was observed by microscopy. ImageJ software calculated the extent of wound healing and the healing rate of the cell lines {wound healing rate at a given time = [(the initial wound area-48 h wound area)] *100%/initial wound area}.

### 2.8 Statistical analysis

GraphPad Prism 8.0 statistical software was used to analyze the data. Measures were expressed as mean standard deviation, with *p* < 0.05 indicating a statistically significant difference.

## 3 Results

### 3.1 Genetic variation in TRP channel-related genes

**FIGURE 1:**
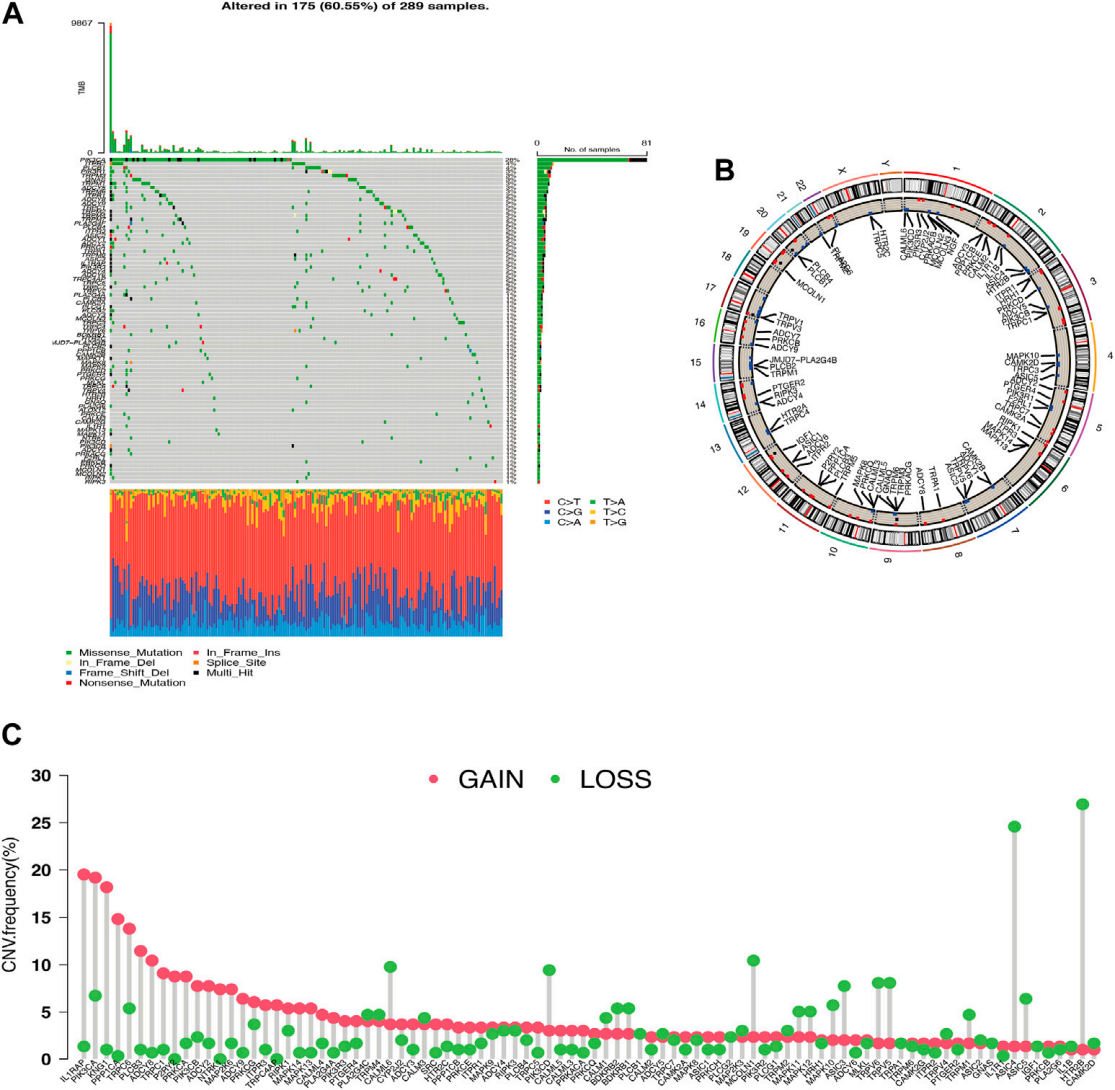
Genetic variation profile of Transient Receptor Potential channel-related gene (TRG) **(A)** Gene mutations carried in cervical cancer samples. **(B)** Copy number variation altered loci on chromosomes for TRG. **(C)** Frequency of copy number variation in TRG.

Thirty-one of the 289 cervical carcinoma samples had TRP mutations, mostly missense mutations. The most frequent mutation was found in PIK3CA (Figure 1A). CNV alterations were prevalent in most TRP channel-related genes (TRG), with most alterations concentrated in copy number amplification deletions, but some TRG deletions were more frequent (Figure 1C). The CNV distribution of TRG on the chromosomes was mapped (Figure 1B). TRG with higher amplification frequencies were found to have higher mRNA expression levels in cancerous tissues than in normal tissues, such as ILIRAP, PIK3CA, PPPICA, and PLCB3, suggesting that TRG may be tumor heterogeneous in normal versus cancerous cervical samples (Supplementary Figure S1A).

### 3.2 Integrated analysis of biological behavior and immune infiltration of TRP

**FIGURE 2:**
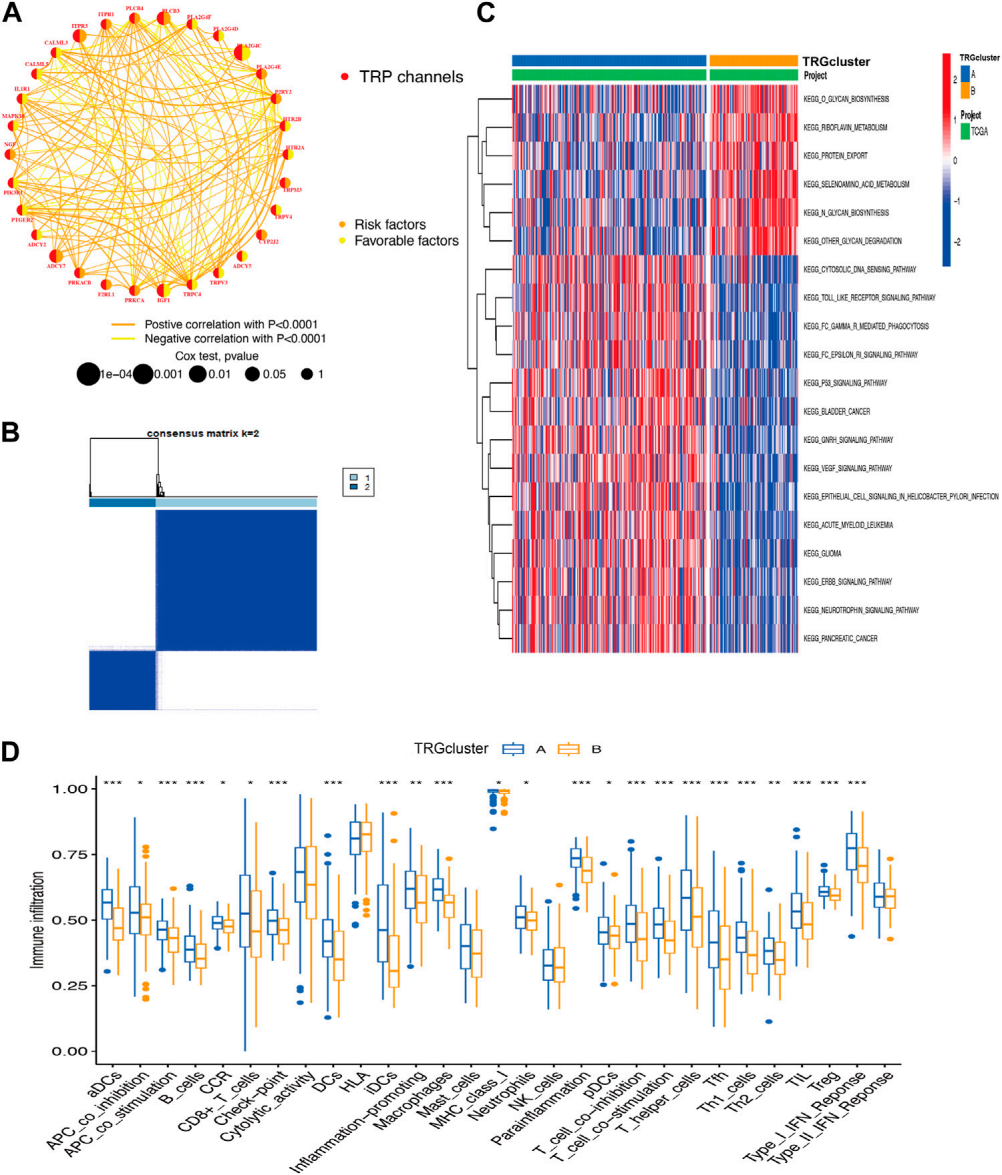
Integrated analysis of biological behaviors and immune infiltration of TRP **(A)** TRG correlation analysis **(B)** TRG-based consensus matrixes of cervical cancer samples (k = 2) **(C)** GSVA displaying the biological behaviors’ activation state in TRG clusters A and B **(D)** the clusters A and B for the abundance of immune infiltration (*p < 0.05, **p < 0.01, ***p < 0.001).

**FIGURE 3:**
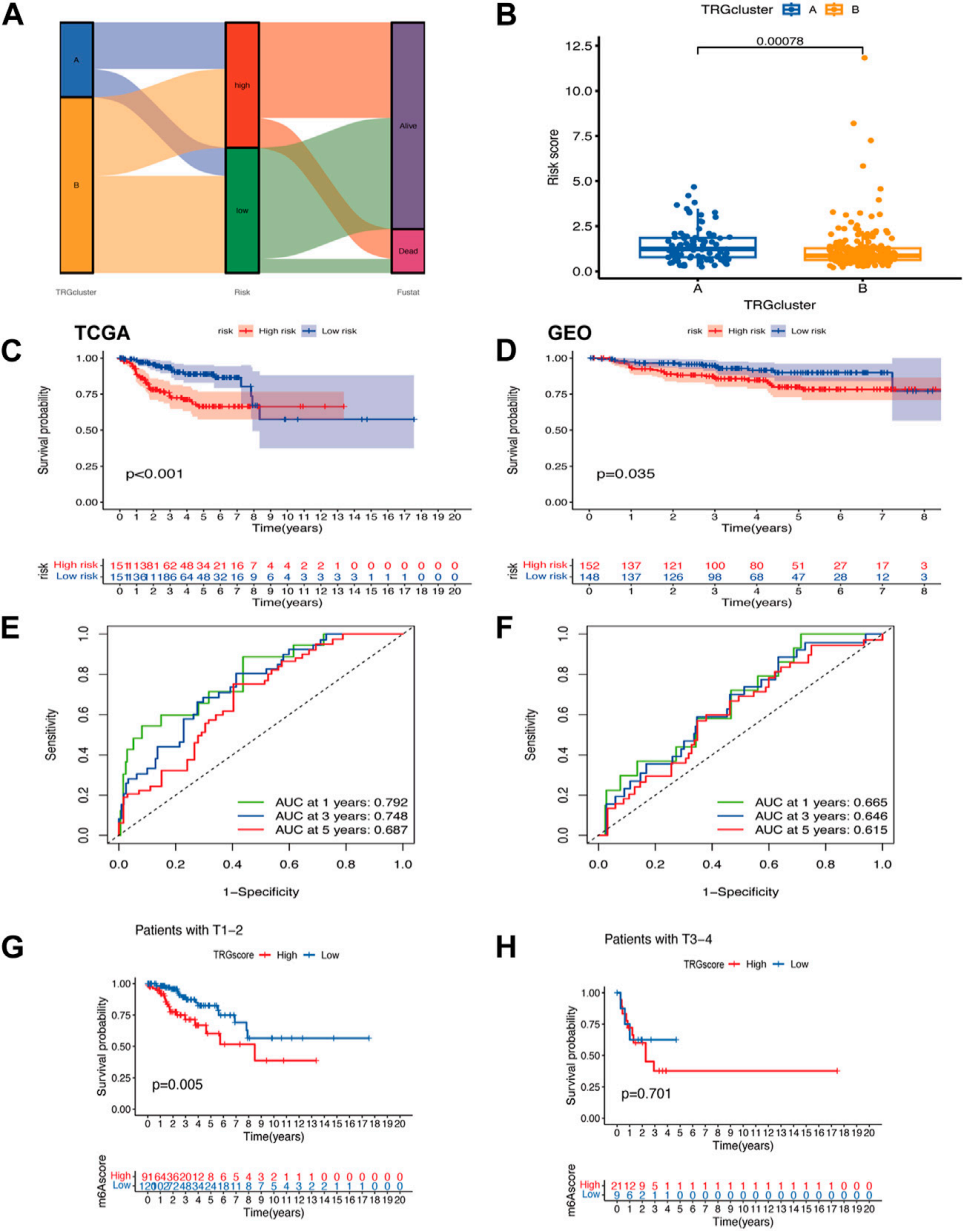
Modeling of TRGs risk signature **(A)** Sankey diagram showing the distribution of sample TRG clusters, TRGs clusters, and prognostic information of patients **(B)** Differences in risk scores between TRG clusters **(C,D)** Kaplan-Meier curves showing the overall survival analysis of TRGs in TCGA (test group) **(C)** versus GEO (validation group) **(D)**. **(E,F)** ROC curves test the effect of the model in the TCGA **(E)** and GEO **(F)** datasets. **(G,H)** Analysis of differences between high and low-risk groups in clinical tumor infiltration grading.

A total of 30 TRG associated with prognosis were screened, these genes are named transient receptor potential channel prognosis-related genes (TRGs) (*p* < 0.05) (Figure 2A). The results revealed that the same prognostic influencing genes were mostly positively correlated, such as a significant positive correlation between the benign prognostic genes TRPC4, TRPV3, and TRPV4. To further explore the biological behavior of TRG in cervical cancer, the “Consensus Cluster Plus package” was used to divide TRG into two clusters based on their expression (Figure 2B; Supplementary Figure S1B). The Cluster A group is a high-risk group, and most of the pathway-related enrichments are positively associated with immune signaling pathways, such as Toll-like receptor signaling pathway, Fc epsilon RI signaling pathway, and p53 signaling pathway (Figures 2C, 3B). Correspondingly in the immunoassay, the level of immune infiltration was generally higher in the high-risk Cluster A group compared to the Cluster B group. These included some immunosuppressive cells such as CD8^+^ T cells, regulatory T cells (Tregs), macrophages, and mast cells (Figure 2D).

### 3.3 TRGs risk signature development and identification

A visualization of the TRG grouping and TRGs grouping of cervical cancer samples relative to the prognostic information of patients was presented in a sankey diagram (Figure 3A). The key genes (PLA2G4C, IL1B, ADCY1, PRKCB, and TRPC4AP) were screened by Lasso regression for variables. A prognostic signature was constructed based on the expression of these five genes and the patients in the sample were classified into high and low risk groups. Their risk score = (PLA2G4C*-0.189099697516017) + (IL1B*0.254146497223738) + (ADCY1*0.371333082335357) + (PRKCB*-0.461389785536792) + (TRPC4AP*0.6989900211777). Using survival estimates based on the optimal cutoff expression value for each gene, results showed that the high-risk group score group had a poorer prognosis (*p* = 0.001) and that the number of deaths increased with increasing risk score and ROC curves to evaluate the effect of the signature. The results of the analysis in the validation cohort (GEO cohort) were also as expected (Figures 3C–F; Supplementary Figures S2A–F). Combined with the clinical traits of the patients, a worse prognosis was found with a higher risk score in the T_1_-T_2_ subgroup (*p* = 0.005) (Figures 3G, H).

### 3.4 Functional enrichment analysis of TRGs

**FIGURE 4:**
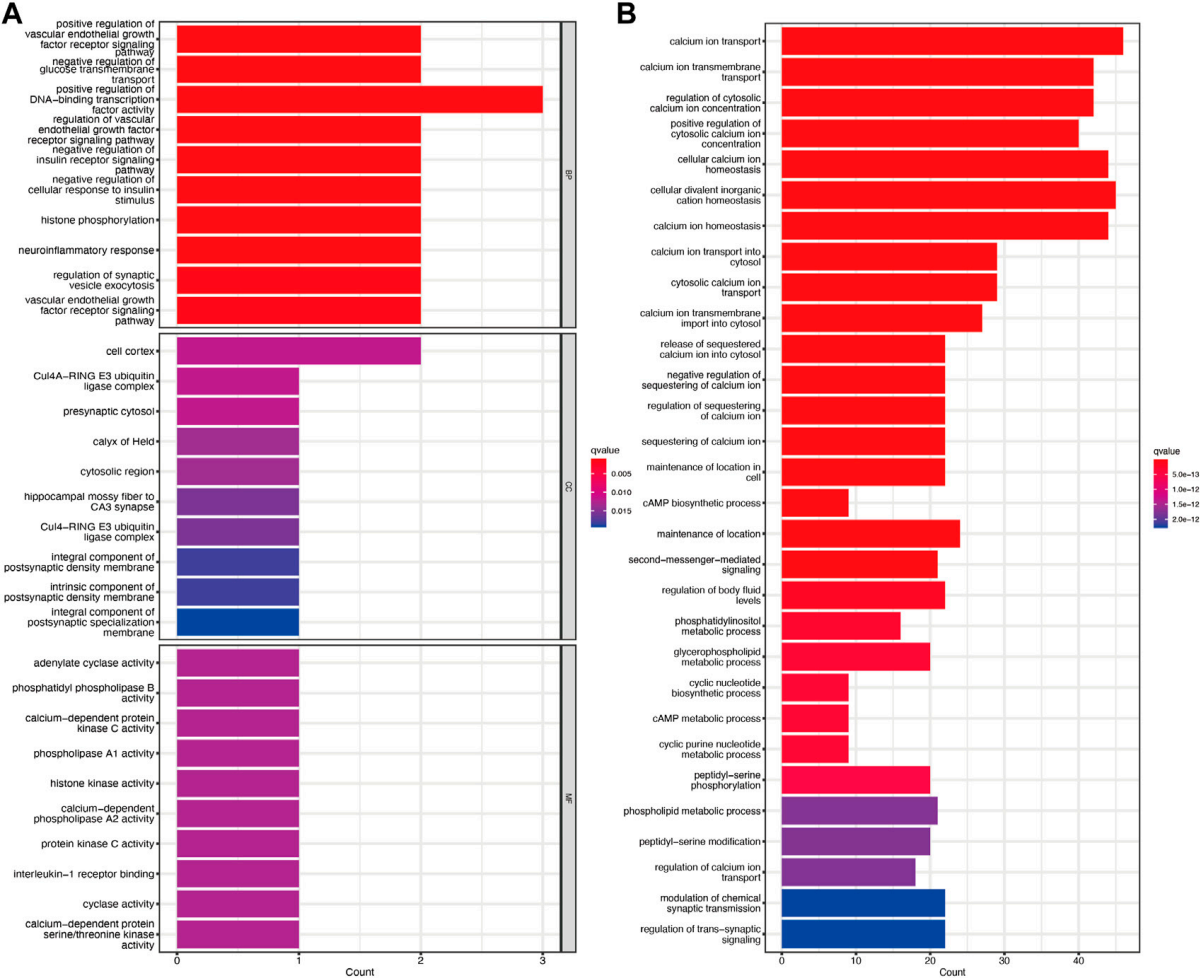
Functional enrichment analysis of TRGs **(A)** GO enrichment analysis of TRGs in cervical cancer **(B)** KEGG enrichment analysis of TRGs in cervical cancer.

GO enrichment analysis showed that the molecular function, biological process, and cellular component of TRGs were mostly gathered in information transfer, such as positive regulation of DNA-binding transcription factor activity, presynaptic cytosol, and calcium-dependent protein kinase C activity (Figure 4A). Pathway enrichment analyses revealed that TRGs were closely associated with calcium ion and metabolic pathways such as calcium ion transport, calcium ion transmembrane transport, and regulation of cytosolic calcium ion concentration and cAMP metabolic process (Figure 4B).

### 3.5 TRGs risk score combined with tumor mutational burden to predict prognosis

**FIGURE 5:**
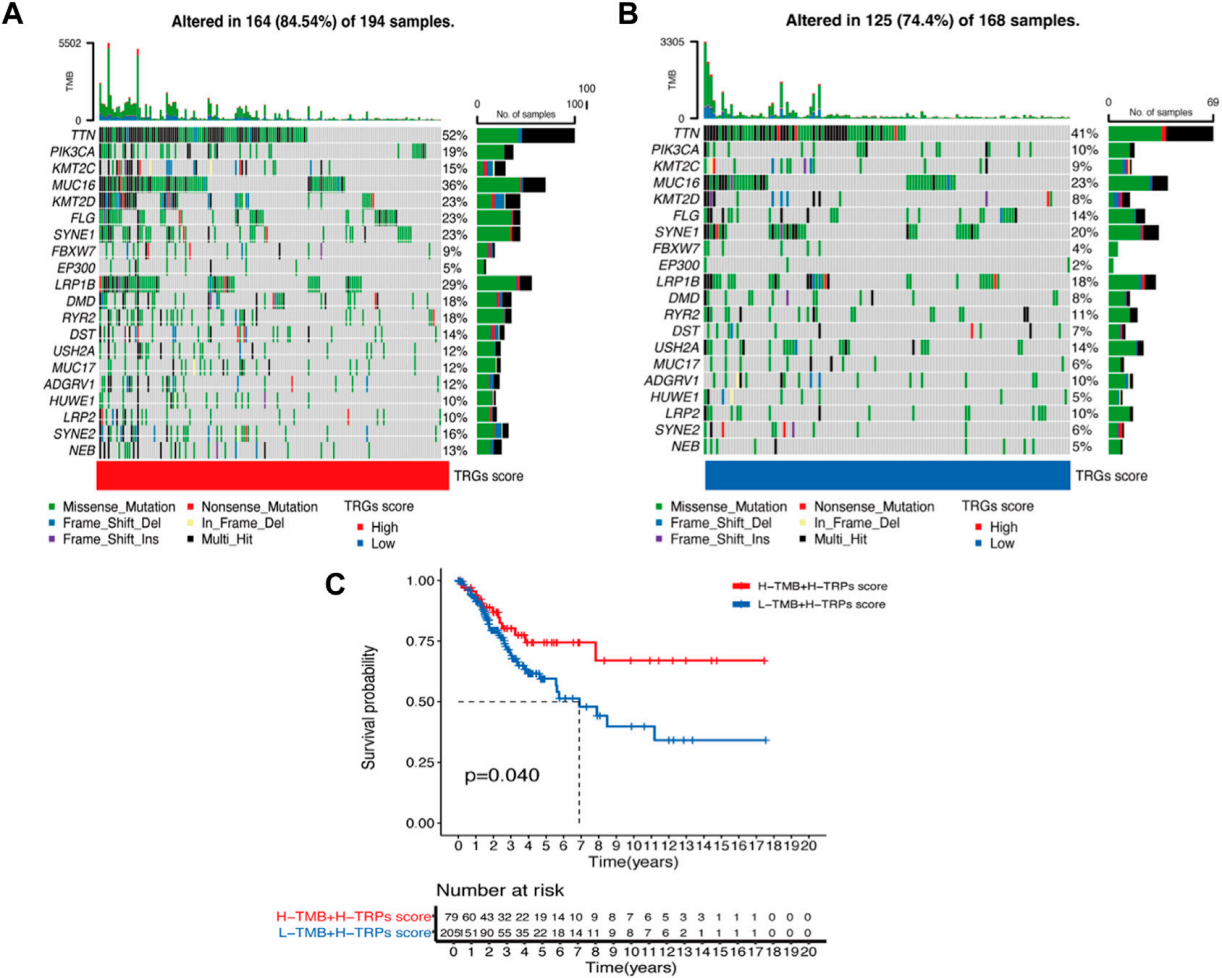
Waterfall plot of mutation frequencies in the high and low-risk groups of TRGs **(A,B)** Analysis of the difference in mutation frequency between the high-risk group **(A)** and the low-risk group **(B)**. **(C)** Kaplan-Meier curves show the overall survival differences between the different TMB subgroups.

We analyzed differences in the genes with the highest frequency of the top 20 mutations in somatic mutations in the different risk groups. The high-risk group had a higher proportion of mutations compared to the low-risk group (Figures 5A, B). The highest mutation frequencies were found in TTN, PIK3CA, and KMT2C. The most common mutation type was also missense mutation. In the prognostic analysis in combination with TMB, the high TMB and high-risk score groups had a better prognosis and *vice versa*, which may provide new ideas for immunotherapy (Figure 5C).

### 3.6 The great potential of the TRGs risk signature for therapy

**FIGURE 6:**
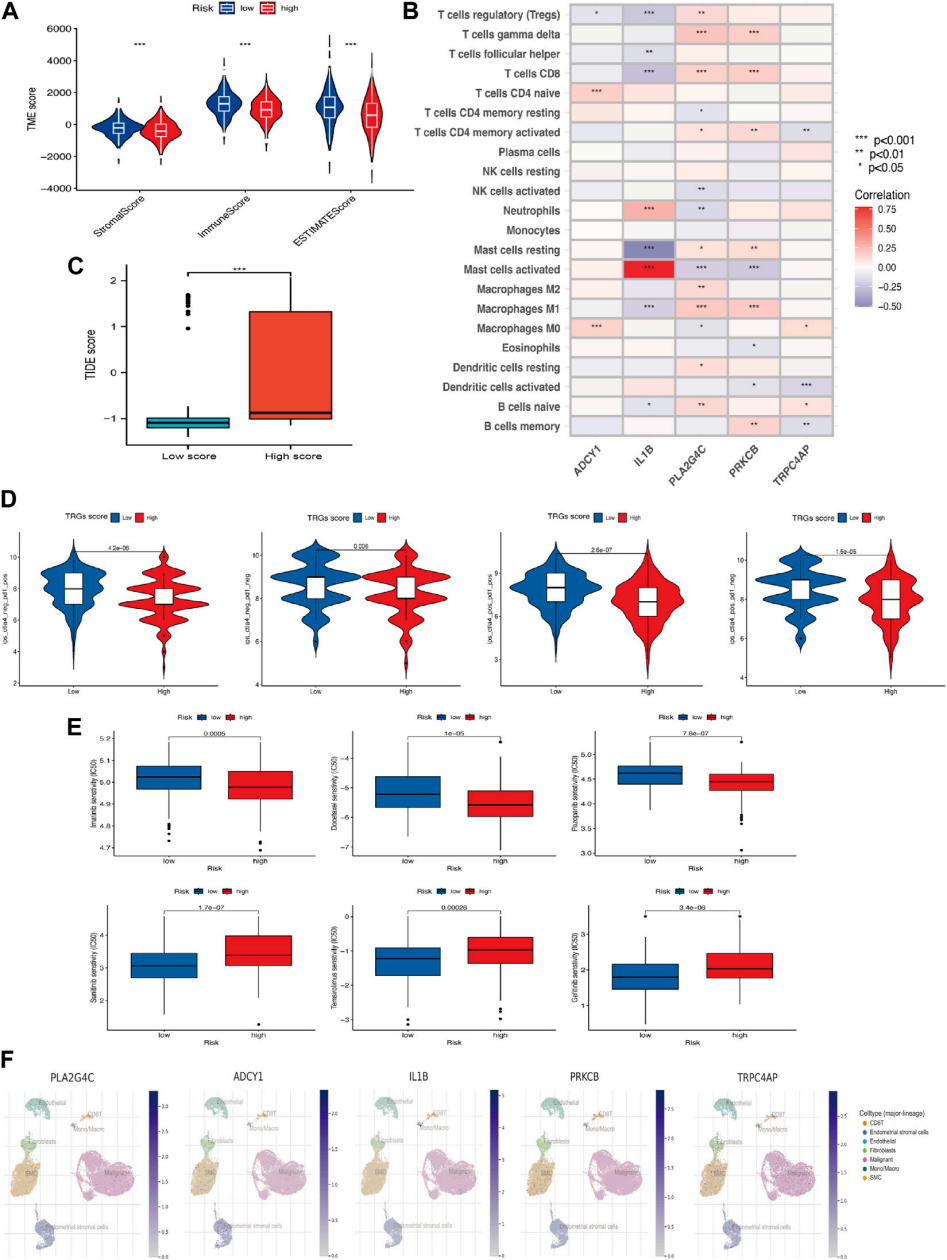
Association of TRGs signature in immunotherapy and targeted therapy **(A)** Differences in the tumor microenvironment between high-risk and low-risk groups. **(B)** Five prognosis-related TRGs and immune cell infiltration are correlated. **(C)** TIDE scores among different TRGs risk groups. **(D)** Differences in TRGs between TRGs risk groups. There were significant differences between the high- and low-risk groups. **(E)** Sensitivity analysis of drugs (Pazopanib, Imatinib, Docetaxel, Sunitinib, Temsirolimus & Gefitinib) between high and low-risk groups. **(F)** UMAP visualization of five model key genes in essential cervical cancer cell subpopulations (GSE168652). (*p < 0.05, **p < 0.01, and ***p < 0.001).

Immune scores, Stromal scores, and ESTIMATE scores were assessed between the different risk groups for comparison (Figure 6A) and there were differences in these aspects between the high and low-risk groups, with the high-risk group having lower scores. In addition, TRGs were correlated to varying degrees in most immune cells (Figure 6B). The TIDE score was used to evaluate the response to treatment with ICI in the different analyzed high and low-risk groups (Figure 6C). Given the differences in mutation and immune infiltration between the high and low-risk groups, patients were further assessed for the possibility of applying immune checkpoint inhibitors (ICI) by analyzing the association between immune cell proportion score (IPS) and risk signature. The high-risk group was also less effective in the IPS, IPS-PD1/PD-L1/PD-L2, IPS-CTLA4, and IPS-PD1/PD-L1/PD-L2 + CTLA4 subgroups of treatment assessment (Figure 6D). The “pRRophetic package” was used to determine the effect of risk score on drug sensitivity. Common cervical cancer targeted drugs such as Sunitinib, Temsirolimus and Gefitinib are more effective in high-risk groups (Figure 6E). A single-cell study of gene expression in the tumor microenvironment, encompassing immune cells, stromal cells, malignant cells, and functional cells, revealed that the key gene in the model was TRPC4AP, which was more widely distributed in malignant cells (Figure 6F).

### 3.7 TRP channel inhibitors’ impact on cervical cancer

**FIGURE 7:**
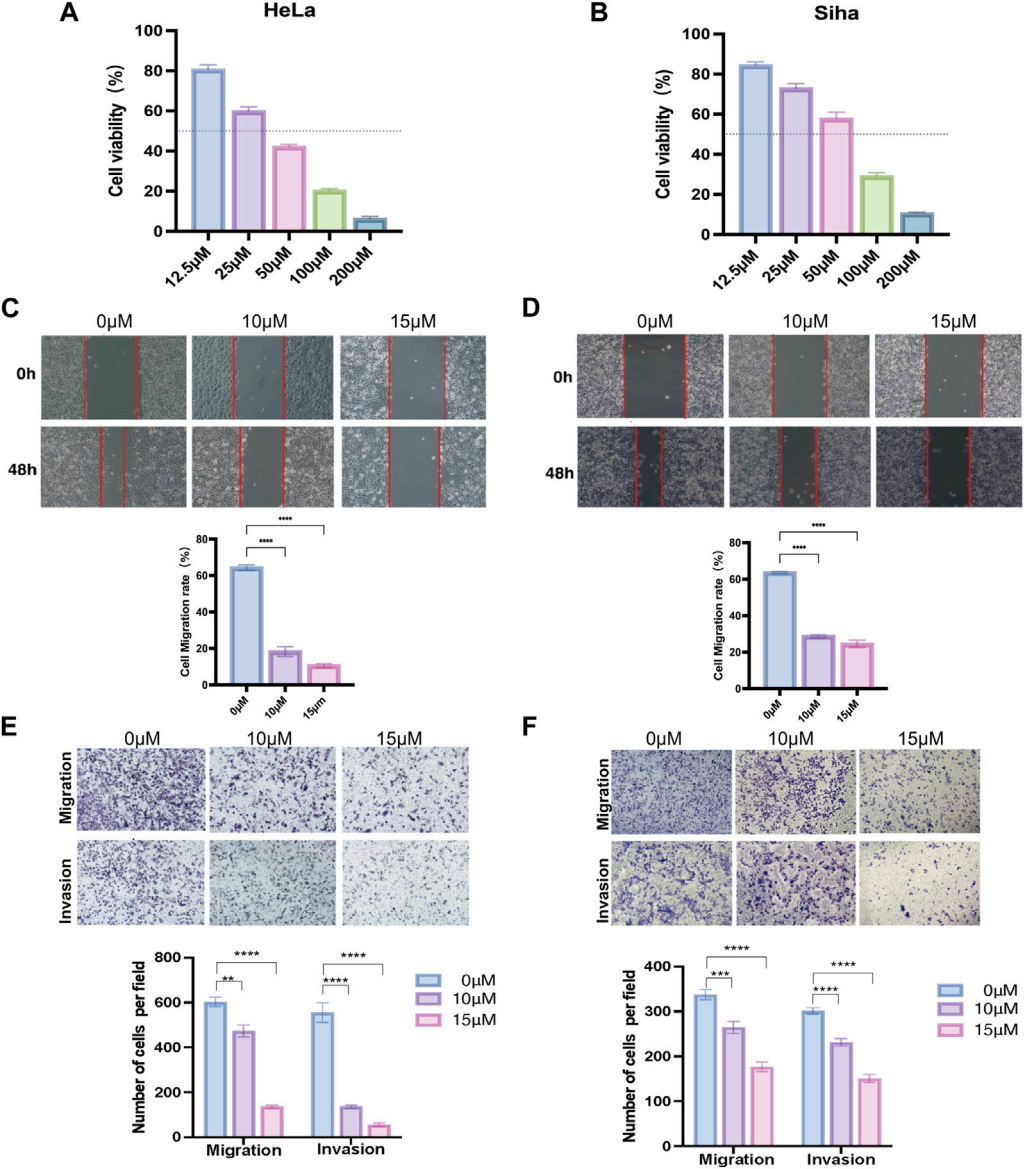
Effect of TRP channel inhibitors on cervical cancer cells verified by *in vitro* experiments **(A,B)** Effect of TRP channel inhibitors on the viability of HeLa **(A)** and Siha **(B)** cervical cancer cells **(C,D)** Scratch assay to detect the migration ability of HeLa **(C)** and Siha **(D)** cells. **(E,F)** Transwell assay to detect the migration and invasion ability of HeLa **(E)** and Siha **(F)** cells. (**p < 0.01, and ***p < 0.001, ****p < 0.0001).

The proliferation capacity of cervical cancer cells (HeLa and Siha) was assessed by the CCK-8 assay after 24 h of the action of various doses of TRP channel inhibitors to evaluate the influence of the TRP channel on the proliferation of cervical cancer cells. The development of cancer cells was suppressed by the pathway inhibitors in a dose-dependent manner, as illustrated in Figures 7A, B. At 36.01 and 54.20 μM concentration inhibitors, HeLa and Siha cells displayed around 50% suppression of cell proliferation, respectively. According to the results, HeLa and Siha cells’ wound healing rates tended to decline with increasing inhibitor dose (Figures 7C, D). In comparison to the control group, the number of cervical cancer cells that migrated and invaded within 24 h reduced with increasing dosages of the inhibitor, according to the transwell assay (Figures 7E, F).

## 4 Discussion

Cervical cancer, a common and dangerous malignancy affecting women, is a complex disease regulated by multiple genes. Early symptoms are often subtle and diverse, making screening and physical examinations crucial (Liu et al., 2023). Patients may neglect the disease because it cannot be detected without cervical cancer screening and physical examination. After all, the disease’s early symptoms are uncommon and its causes are varied (Gottschlich et al., 2023). According to statistics, the prevalence of cervical cancer is rising, therefore it’s critical to raise women’s knowledge of the disease and do the essential cervical cancer screening to detect the disease early and begin treatment (Siegel et al., 2023).

There is growing evidence that TRP channels play a role in the development and progression of cervical cancer. One of the most well-studied TRP channels in cervical cancer is TRPV1. TRPV1 is overexpressed in cervical cancer tissues and cell lines and is associated with cervical cancer cell proliferation, migration, and invasion (Sánchez-Sánchez et al., 2015; Wang et al., 2022). In addition, through the activation of the β-catenin signaling pathway, TRPM4 has been demonstrated to promote cervical cancer cell proliferation and invasion (Armisén et al., 2011). Other TRP channels have also been associated with cervical cancer. In cervical cancer, TRPM7 expression regulated miR-543-mediated cell cycle arrest, increased apoptosis *in vitro*, and inhibited tumor growth *in vivo* (Liu et al., 2019). Similarly, TRPM8 binding to Rap1 inhibited the adhesion of cervical cancer cells (Chinigò et al., 2022). Targeting TRP channels in cervical cancer is shown promising as a therapeutic target. Several TRP channel antagonists and agonists have been developed and tested undergoing preclinical studies testing (De La Chapa et al., 2019; Chai et al., 2022; Chen et al., 2023; Neuberger et al., 2023). However, there is a lack of an evaluation strategy based on the transient receptor potential channels to predict patient prognosis individually. Therefore, this study comprehensively analyzed the tumor microenvironment, immune infiltration, and potential impact of immunotherapy of TRG in cervical cancer to explore its intrinsic linkage, maximize the anti-tumor effect of TRG, combine chemotherapy, radiotherapy and immunotherapy, improve the efficacy of anti-tumor therapy.

To establish a systematic multi-gene biomarker signature, Cox regression screening for TRG was followed by Lasso regression to establish a TRGs-based prognostic signature. The overall survival curve predicted that the high-risk group had a poor clinical outcome, while in comparison, patients with lower risk scores had a good prognosis, and the AUC in the ROC curve largely explains the reliability and applicability of this signature. Analysis of its biological behavior revealed that TRGs in the KEGG pathway are strongly associated with calcium signaling. This suggests that the genes screened in this signature play a key role in the transient receptor potential. The calcium signaling pathway is a source of energy on which a variety of cells rely for survival. Calcium imbalance is associated with tumor progressions, such as proliferation, invasion, and metastasis. Tumour immune dysfunction and rejection may be present due to higher TIDE scores in the high-risk group. The sensitivity to PD-1 and CTLA-4 inhibitors alone or in combination was found to be lower in the high-risk group than in the low-risk group in the IPS analysis. TIDE and IPS analyses suggest that immunotherapy, especially PD-1 and CTLA-4 inhibitors, is not recommended for patients in the high-risk group, but patients in the high-risk group with higher mutation loads may have better outcomes with immunosuppressive therapy. For patients who are not sensitive to immunological drugs, the treatment schedule should be changed in time, or targeted drugs may be used to improve the prognosis of patients to a greater extent. It has been reported that Gefitinib, as an inhibitor of EGFR, attenuates the effect of transient receptor potential melastatin 7 (TRPM7) on the migration and proliferation of vascular smooth muscle cells stimulated by epidermal growth factor. Therefore, in-depth studies on the pathogenesis of TRGs in cervical cancer can provide new ideas for the development of new molecularly targeted drugs, and have great clinical translational value for the research of TRP channels in oncology drugs, which is still a gap in the development of targeted drug therapy against cervical cancer.

We attempted to apply the effect of a TRP channel inhibitor 1-[β-(3-(4-Methoxyphenyl)propoxy)-4-methoxyphenethyl]-1H-imidazole, a selective inhibitor of receptor-mediated Ca^2+^ inward flow and voltage-gated Ca^2+^ inward flow, currently mainly as a TRPC channel blocker, which shows the effect on cervical cancer cell growth. Notably, this drug promotes Pyk2 upregulation, hinders glioma progression and enhances focal adhesion formation by inhibiting TRPC4AP (Ding et al., 2006; Cheng et al., 2011). In gastric cancer cells, this inhibitor has demonstrated the ability to block endogenous TRPC6 channels, leading to cell cycle arrest in the G_2_/M phase and inhibiting cell growth (Cai et al., 2009).

Our research intends to develop a prognostic risk model that would offer feasible options for prognosis screening and targeted therapy of CC patients. However, there are still some limitations of this study worth mentioning. The validation of the signature is limited to the data and the ROC is not at an optimal value due to the short follow-up time of the GEO data cohort. Therefore, we need to collect real clinical samples in subsequent studies to verify the accuracy of this signature in predicting patient prognosis. In addition, whether TRP channel inhibitors can be applied to patients with cervical cancer, and the connection between the selected TRP channel key factors and cervical cancer, more research on the molecular mechanism is needed. *In vivo* and *in vitro* experiments will further reveal how the TRP channel participates in the development process of cervix cancer. Overall, Our findings may enable stratification of CC patients with high risk, poor prognosis, and variable treatment sensitivity based on the risk signature, thereby improving clinical outcomes for CC patients.

## Data Availability

Publicly available datasets were analyzed in this study. This data can be found here: https://tcga-data.nci.nih.gov/tcga/ and https: //www.ncbi.nlm.nih.gov/geo/ ID:GSE44001.

## Abbreviations

CC, cervical cancer; TRP, transient receptor potential; TRG, channel-related genes; TRGs, transient receptor potential channel prognosis-related genes; TCGA, The Tumor Genome Atlas; GEO, The Gene Expression Omnibus; SNV, single nucleotide variation; CNV, Copy number variation; LASSO, least absolute shrinkage and selection operator; Tregs, regulatory T cells; ICI, immune checkpoint inhibitors; IPS, immune cell proportion score; TRPM7, Transient receptor potential melastatin.

## References

[B1] Armisén R. Marcelain K. Simon F. Tapia J. C. Toro J. Quest A. F. (2011). TRPM4 enhances cell proliferation through up-regulation of the β-catenin signaling pathway. J. Cell Physiol. 226 (1), 103–109. 10.1002/jcp.22310 20625999

[B2] Baral P. Umans B. D. Li L. Wallrapp A. Bist M. Kirschbaum T. (2018). Nociceptor sensory neurons suppress neutrophil and γδ T cell responses in bacterial lung infections and lethal pneumonia. Nat. Med. 24 (4), 417–426. 10.1038/nm.4501 29505031 PMC6263165

[B3] Cai R. Ding X. Zhou K. Shi Y. Ge R. Ren G. (2009). Blockade of TRPC6 channels induced G2/M phase arrest and suppressed growth in human gastric cancer cells. Int. J. Cancer 125 (10), 2281–2287. 10.1002/ijc.24551 19610066

[B4] Cai R. Wang L. Liu X. Michalak M. Tang J. Peng J. B. (2021). Auto-inhibitory intramolecular S5/S6 interaction in the TRPV6 channel regulates breast cancer cell migration and invasion. Commun. Biol. 4 (1), 990. 10.1038/s42003-021-02521-3 34413465 PMC8376870

[B5] Caterina M. J. Julius D. (2001). The vanilloid receptor: a molecular gateway to the pain pathway. Annu. Rev. Neurosci. 24, 487–517. 10.1146/annurev.neuro.24.1.487 11283319

[B6] Caterina M. J. Pang Z. (2016). TRP channels in skin biology and pathophysiology. Pharm. (Basel) 9 (4), 77. 10.3390/ph9040077 PMC5198052 27983625

[B7] Chai X. N. Ludwig F. A. Müglitz A. Gong Y. Schaefer M. Regenthal R. (2022). A pharmacokinetic and metabolism study of the TRPC6 inhibitor SH045 in mice by LC-MS/MS. Int. J. Mol. Sci. 23 (7), 3635. 10.3390/ijms23073635 35408998 PMC8998618

[B8] Chen L. Mao M. Liu D. Liu W. Wang Y. Xie L. (2023). HC067047 as a potent TRPV4 inhibitor repairs endotoxemia colonic injury. Int. Immunopharmacol. 116, 109648. 10.1016/j.intimp.2022.109648 36706595

[B9] Cheng J. S. Shu S. S. Kuo C. C. Chou C. T. Tsai W. L. Fang Y. C. (2011). Effect of diindolylmethane on Ca(2+) movement and viability in HA59T human hepatoma cells. Arch. Toxicol. 85 (10), 1257–1266. 10.1007/s00204-011-0670-9 21409406

[B10] Chinigò G. Grolez G. P. Audero M. Bokhobza A. Bernardini M. Cicero J. (2022). TRPM8-Rap1A interaction sites as critical determinants for adhesion and migration of prostate and other epithelial cancer cells. Cancers (Basel) 14 (9), 2261. 10.3390/cancers14092261 35565390 PMC9102551

[B11] Cohen P. A. Jhingran A. Oaknin A. Denny L. (2019). Cervical cancer. Lancet 393 (10167), 169–182. 10.1016/S0140-6736(18)32470-X 30638582

[B12] De La Chapa J. Valdez M. Ruiz F. Gonzales K. Mitchell W. McHardy S. F. (2019). Synthesis and SAR of novel capsazepine analogs with significant anti-cancer effects in multiple cancer types. Bioorg Med. Chem. 27 (1), 208–215. 10.1016/j.bmc.2018.11.040 30528162

[B13] Ding Y. Robbins J. Fraser S. P. Grimes J. A. Djamgoz M. B. (2006). Comparative studies of intracellular Ca2+ in strongly and weakly metastatic rat prostate cancer cell lines. Int. J. Biochem. Cell Biol. 38 (3), 366–375. 10.1016/j.biocel.2005.07.009 16300989

[B14] Engebretsen S. Bohlin J. (2019). Statistical predictions with glmnet. Clin. Epigenetics 11 (1), 123. 10.1186/s13148-019-0730-1 31443682 PMC6708235

[B15] Fattori V. Zaninelli T. H. Ferraz C. R. Brasil-Silva L. Borghi S. M. Cunha J. M. (2022). Maresin 2 is an analgesic specialized pro-resolution lipid mediator in mice by inhibiting neutrophil and monocyte recruitment, nociceptor neuron TRPV1 and TRPA1 activation, and CGRP release. Neuropharmacology 216, 109189. 10.1016/j.neuropharm.2022.109189 35820471

[B16] Geeleher P. Cox N. Huang R. S. (2014). pRRophetic: an R package for prediction of clinical chemotherapeutic response from tumor gene expression levels. PLoS One 9 (9), e107468. 10.1371/journal.pone.0107468 25229481 PMC4167990

[B17] Gottschlich A. Payne B. A. Trawin J. Albert A. Jeronimo J. Mitchell-Foster S. (2023). Community-integrated self-collected HPV-based cervix screening in a low-resource rural setting: a pragmatic, cluster-randomized trial. Nat. Med. 29 (4), 927–935. 10.1038/s41591-023-02288-6 37037880

[B18] Leek J. T. Johnson W. E. Parker H. S. Jaffe A. E. Storey J. D. (2012). The sva package for removing batch effects and other unwanted variation in high-throughput experiments. Bioinformatics 28 (6), 882–883. 10.1093/bioinformatics/bts034 22257669 PMC3307112

[B19] Lehen kyi V. Flourakis M. Skryma R. Prevarskaya N. (2007). TRPV6 channel controls prostate cancer cell proliferation via Ca(2+)/NFAT-dependent pathways. Oncogene 26 (52), 7380–7385. 10.1038/sj.onc.1210545 17533368

[B20] Li W. Chen X. Riley A. M. Hiett S. C. Temm C. J. Beli E. (2017). Long-term spironolactone treatment reduces coronary TRPC expression, vasoconstriction, and atherosclerosis in metabolic syndrome pigs. Basic Res. Cardiol. 112 (5), 54. 10.1007/s00395-017-0643-0 28756533 PMC5534204

[B21] Li X. Zhang Q. Fan K. Li B. Li H. Qi H. (2016). Overexpression of TRPV3 correlates with tumor progression in non-small cell lung cancer. Int. J. Mol. Sci. 17 (4), 437. 10.3390/ijms17040437 27023518 PMC4848893

[B22] Liu C. Li X. Huang Q. Zhang M. Lei T. Wang F. (2023). Single-cell RNA-sequencing reveals radiochemotherapy-induced innate immune activation and MHC-II upregulation in cervical cancer. Signal Transduct. Target Ther. 8 (1), 44. 10.1038/s41392-022-01264-9 36710358 PMC9884664

[B23] Liu X. Gan L. Zhang J. (2019). miR-543 inhibites cervical cancer growth and metastasis by targeting TRPM7. Chem. Biol. Interact. 302, 83–92. 10.1016/j.cbi.2019.01.036 30710498

[B24] Mayakonda A. Lin D. C. Assenov Y. Plass C. Koeffler H. P. (2018). Maftools: efficient and comprehensive analysis of somatic variants in cancer. Genome Res. 28 (11), 1747–1756. 10.1101/gr.239244.118 30341162 PMC6211645

[B25] Moore C. Liedtke W. B.: Frontiers in neuroscience osmomechanical-sensitive TRPV channels in mammals. In: Neurobiology of TRP channels. edn. Edited by Emir T. L. R. Boca Raton (FL): CRC Press/Taylor & Francis © 2018 by Taylor & Francis Group, LLC.; 2017: 85–94. 29356489

[B26] Negri S. Faris P. Berra-Romani R. Guerra G. Moccia F. (2019). Endothelial transient receptor potential channels and vascular remodeling: extracellular Ca(2 +) entry for angiogenesis, arteriogenesis and vasculogenesis. Front. Physiol. 10, 1618. 10.3389/fphys.2019.01618 32038296 PMC6985578

[B27] Neuberger A. Oda M. Nikolaev Y. A. Nadezhdin K. D. Gracheva E. O. Bagriantsev S. N. (2023). Human TRPV1 structure and inhibition by the analgesic SB-366791. Nat. Commun. 14 (1), 2451. 10.1038/s41467-023-38162-9 37117175 PMC10147690

[B28] Nilius B. Owsianik G. Voets T. Peters J. A. (2007). Transient receptor potential cation channels in disease. Physiol. Rev. 87 (1), 165–217. 10.1152/physrev.00021.2006 17237345

[B29] Pagano E. Romano B. Cicia D. Iannotti F. A. Venneri T. Lucariello G. (2023). TRPM8 indicates poor prognosis in colorectal cancer patients and its pharmacological targeting reduces tumour growth in mice by inhibiting Wnt/β-catenin signalling. Br. J. Pharmacol. 180 (2), 235–251. 10.1111/bph.15960 36168728 PMC10092658

[B30] Pu J. T. Zhang T. He K. M. Zhang D. G. Teng Z. Y. Wu Y. F. (2022). Transient receptor potential vanilloid 4 promotes the growth of non-small cell lung cancer by regulating Foxp3. Acta Biochim. Pol. 69 (1), 51–57. 10.18388/abp.2020_5614 34995050

[B31] Sánchez-Sánchez L. Alvarado-Sansininea J. J. Escobar M. L. López-Muñoz H. Hernández-Vázquez J. M. Monsalvo-Montiel I. (2015). Evaluation of the antitumour activity of Rinvanil and Phenylacetylrinvanil on the cervical cancer tumour cell lines HeLa, CaSKi and ViBo. Eur. J. Pharmacol. 758, 129–136. 10.1016/j.ejphar.2015.04.003 25864613

[B32] Schnipper J. Kouba S. Hague F. Girault A. Rybarczyk P. Telliez M. S. (2022). The TRPC1 channel forms a PI3K/CaM complex and regulates pancreatic ductal adenocarcinoma cell proliferation in a Ca(2+)-independent manner. Int. J. Mol. Sci. 23 (14), 7923. 10.3390/ijms23147923 35887266 PMC9323718

[B33] Siegel R. L. Miller K. D. Wagle N. S. Jemal A. (2023). Cancer statistics, 2023. CA Cancer J. Clin. 73 (1), 17–48. 10.3322/caac.21763 36633525

[B34] Stumbar S. E. Stevens M. Feld Z. (2019). Cervical cancer and its precursors: a preventative approach to screening, diagnosis, and management. Prim. Care 46 (1), 117–134. 10.1016/j.pop.2018.10.011 30704652

[B35] Sung H. Ferlay J. Siegel R. L. Laversanne M. Soerjomataram I. Jemal A. (2021). Global cancer statistics 2020: GLOBOCAN estimates of incidence and mortality worldwide for 36 cancers in 185 countries. CA Cancer J. Clin. 71 (3), 209–249. 10.3322/caac.21660 33538338

[B36] Wang Z. Dong J. Tian W. Qiao S. Wang H. (2022). Role of TRPV1 ion channel in cervical squamous cell carcinoma genesis. Front. Mol. Biosci. 9, 980262. 10.3389/fmolb.2022.980262 36072430 PMC9444153

[B37] Wilkerson M. D. Hayes D. N. (2010). ConsensusClusterPlus: a class discovery tool with confidence assessments and item tracking. Bioinformatics 26 (12), 1572–1573. 10.1093/bioinformatics/btq170 20427518 PMC2881355

[B38] Wong R. Gong H. Alanazi R. Bondoc A. Luck A. Sabha N. (2020). Inhibition of TRPM7 with waixenicin A reduces glioblastoma cellular functions. Cell Calcium 92, 102307. 10.1016/j.ceca.2020.102307 33080445

[B39] Yu G. Wang L. G. Han Y. He Q. Y. (2012). clusterProfiler: an R package for comparing biological themes among gene clusters. Omics 16 (5), 284–287. 10.1089/omi.2011.0118 22455463 PMC3339379

[B40] Zhang H. Meltzer P. Davis S. (2013). RCircos: an R package for Circos 2D track plots. BMC Bioinforma. 14, 244. 10.1186/1471-2105-14-244 PMC3765848 23937229

