# Supplementary material for: Transient receptor potential channels’ genes forecast cervical cancer outcomes and illuminate its impact on tumor cells
Source: Front Genet. 2024 May 9;15:1391842. doi: 10.3389/fgene.2024.1391842 (PMC11112020; doi:10.3389/fgene.2024.1391842)
Supplement: Supplementary file 1 [file Table1.DOCX]

**Supplementary Material**

**Supplementary Figure 1.**


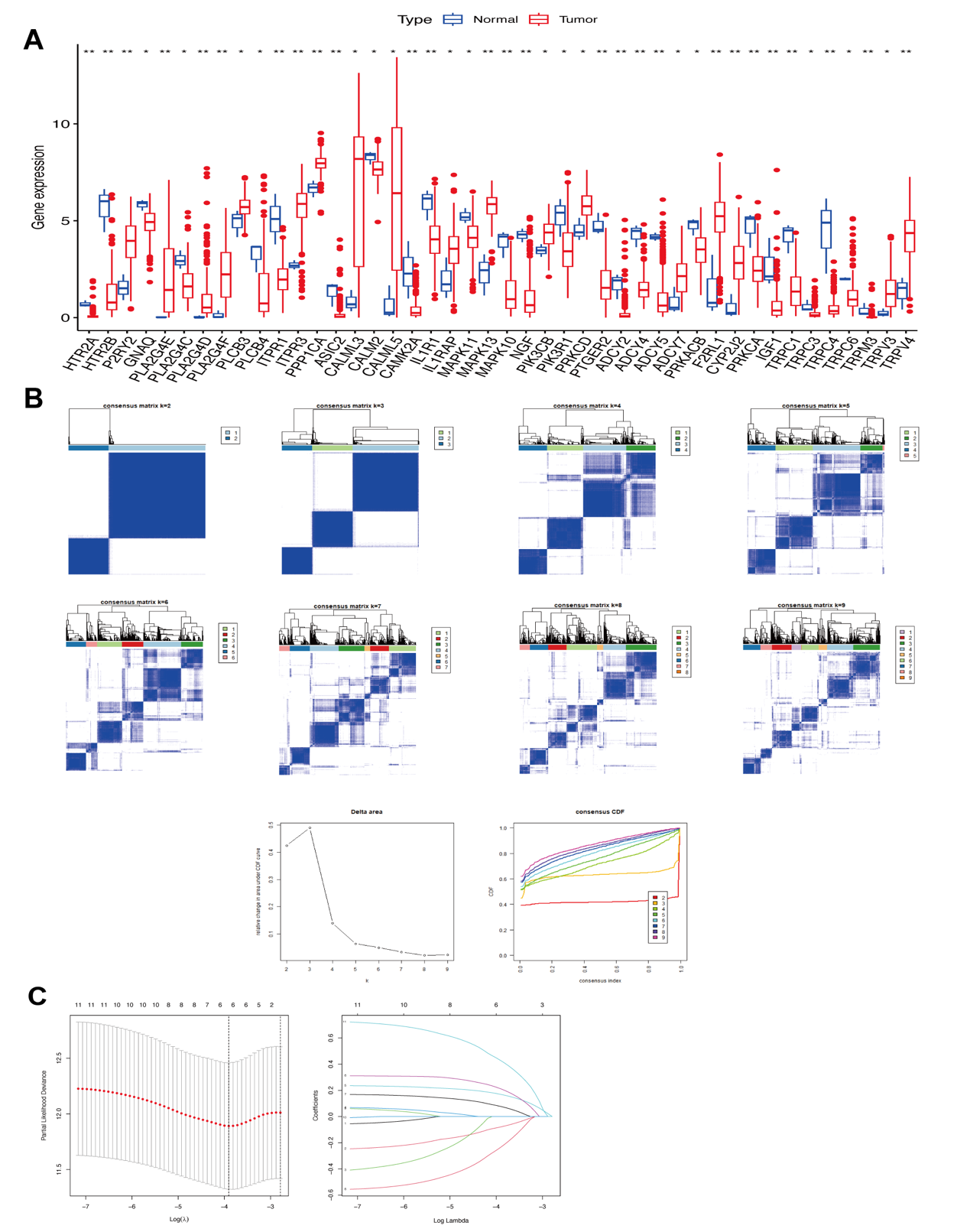


**Supplementary Figure 1.**

(A) Differences in TRG expression in normal and cervical cancer tissue samples (* P < 0.05 and ** P < 0.01). (B) Identification of immune-related gene clustering groups based on immunogenic cell death prognostic gene expression.

**Supplementary Figure 2.**


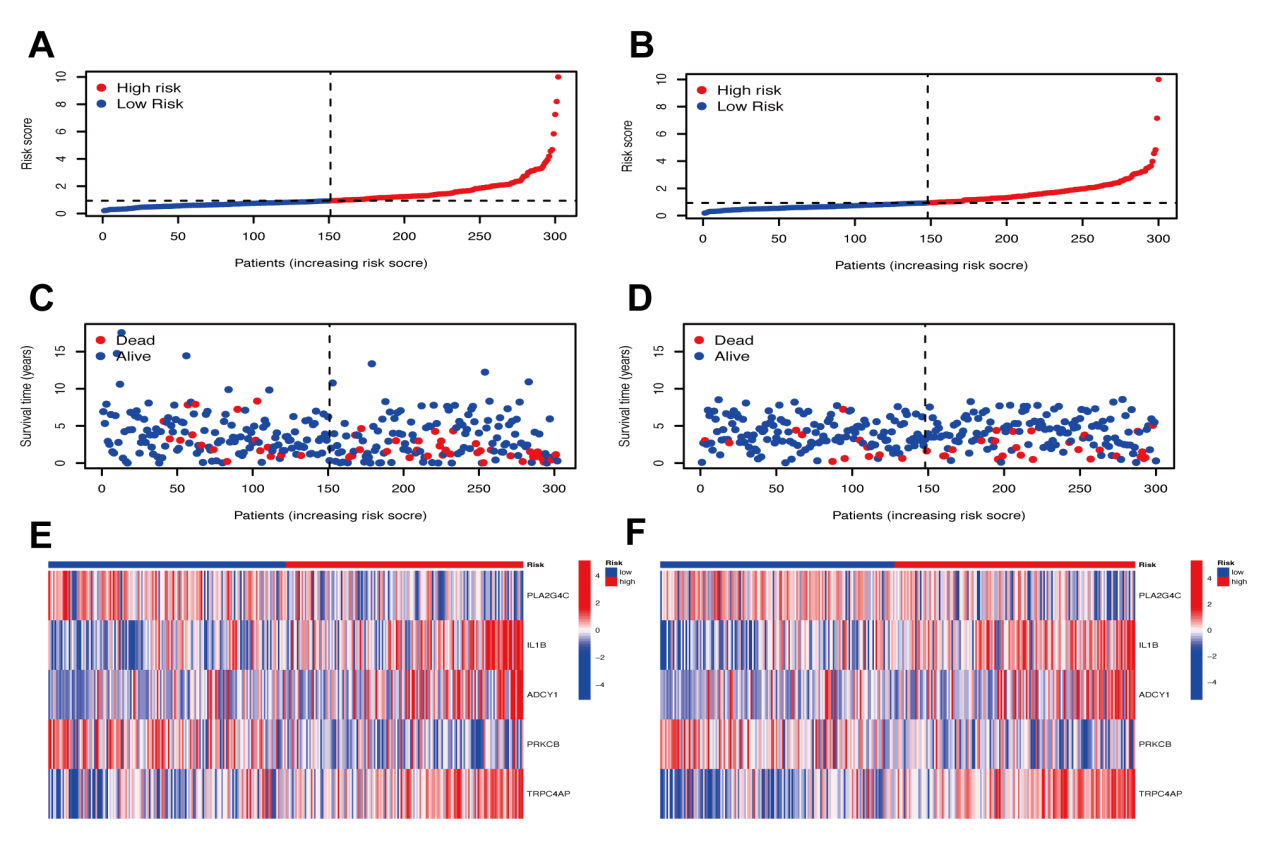


**Supplementary Figure 2.**

(A,B) Distribution of patients in the Cancer Genome Atlas (TCGA) and Gene Expression Omnibus (GEO) cohort experimental groups based on risk scores. (C,D) Survival status of each patient in the TCGA and GEO cohorts. (E,F) Expression of prognostic genes in the high-risk and low-risk groups of the TCGA and GEO cohorts.
